# 282. Occurrence of adverse events and factors related to prognosis in a cohort of patients hospitalized with COVID-19 at University hospital - UNICAMP, Brazil.

**DOI:** 10.1093/ofid/ofac492.360

**Published:** 2022-12-15

**Authors:** Maitê V Luz, Julian F Silva, Hugo D Ceccato, Paulo J Souza, Pedro M E Villar, Paulo R A Mendes, Mariângela R Resende, Mônica C Pereira, Lucieni O Conterno

**Affiliations:** UNICAMP, Campinas, Sao Paulo, Brazil; FAPESP, Campinas, Sao Paulo, Brazil; FAPESP, Campinas, Sao Paulo, Brazil; UNICAMP, Campinas, Sao Paulo, Brazil; UNICAMP, Campinas, Sao Paulo, Brazil; HC-UNICAMP, Campinas, Sao Paulo, Brazil; UNICAMP, Campinas, Sao Paulo, Brazil; UNICAMP, Campinas, Sao Paulo, Brazil; UNICAMP, Campinas, Sao Paulo, Brazil

## Abstract

**Background:**

In Brazil, 30.378.061 cases of COVID were reported and 662.866 deaths up to April 27 of 2022. Hospital infections and other hospital adverse events may increase the risk of death in patients with COVID-19.

**Methods:**

Cohort study that included 650 adult patients hospitalized with a diagnosis of SARS CoV-2 infection at Hospital HC-UNICAMP from March/20 to March/21.

**Results:**

Of the 650 patients included in the study, 139 (21.38%) died. Comparing the patients who died vs those who survived, we observed a statistically significant difference in the occurrence of thromboembolic and vascular events (23% vs 9.8%; OR 1.332; 95%CI 1.12-1.59; p < 0,0001), ICU admission (84.9% vs 39.6%; OR 0.675; 95%CI 0.62-0.74; p< 0.0001) and the occurrence of HI: bloodstream infections (30.2% vs 8.6%; OR 1.62; 95%CI 1.31-1.99; p< 0.0001), VAP (52.5% vs 12.3%; OR 1.882; 95%CI 1.57-2.26; p< 0.0001) and UTI (27.3% vs 7.2%; OR 1.672; 95%CI 1.32-2.11; p< 0.0001). Gram negative bacteria were the most isolated (62.1%), especially *K. pneumoniae*, *A. baumannii* and *P. aeruginosa*, followed by gram positive bacteria (27%) and fungi (13.8%).

When evaluating patients who had thromboembolic events, we observed a statistically significant association with male gender (15.9% vs 7.9%; p 0.003), mean initial D-Dimer values (10,418.00 ng/mL vs 3011.34 ng/ml; p 0.003); acute renal failure (19.1% vs 9.3%; p 0.001) and the occurrence of HI (24.4% vs 7.3%; p< 0.0001).

The following factors associated with ICU admission were identified: *diabetes mellitus* (59.3% vs 40.5%; p< 0.0001); obesity (58.3% vs 41.7%; p 0.003); O2 saturation at admission < 88% (67.8% vs 32.2%); acute renal failure (78.6% vs 21.4%; p< 0.0001) and the occurrence of HI (87.8% vs 12.2%; p< 0.0001).

Logistic regression analysis identified the following variables independently associated with death: age (OR 1,034; CI 1,015-1,052), ICU admission (OR 1,107; CI 1,596-5,868), use of vasoactive drugs (OR 2,93; CI 1,79-4,82), development of acute renal failure (OR 7,756; CI 4,537-13,26), and the occurrence of VAP (OR 2,205; CI 1,227-3,961) (Table 1).

**Table 1:**
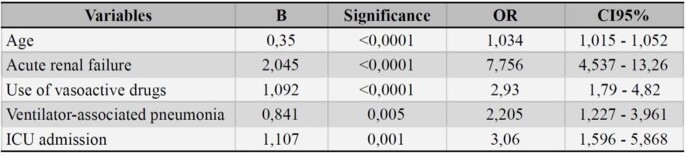
Variables associated to death in Logistic Regression

**Conclusion:**

Adverse events, particularly HI, have an important impact on the evolution of patients with COVID-19, reinforcing the need for prevention and control measures to be optimized as an essential part of the care for these patients.

**Disclosures:**

**Maitê V. Luz, n/a**, FAPESP: Grant/Research Support **Julian F. Silva, MD**, FAPESP: Grant/Research Support **Hugo D. Ceccato, MD**, FAPESP: Grant/Research Support **Mariângela R. Resende, PhD**, FAPESP: Grant/Research Support **Mônica C. Pereira, PhD**, FAPESP: Grant/Research Support **Lucieni O. Conterno, PhD**, FAPESP: Grant/Research Support.

